# CARDIOVASCULAR DISEASE AND FRAILTY IN OLDER ADULTS FROM COLOMBIA

**DOI:** 10.1093/geroni/igad104.2963

**Published:** 2023-12-21

**Authors:** Paola Sosa-Sarmiento, Jose Ocampo-Chaparro, Carlos Reyes-Ortiz

**Affiliations:** Universidad del Valle, Cali, Valle del Cauca, Colombia; Universidad del Valle, Cali, Valle del Cauca, Colombia; Florida A & M University, Tallahassee, Florida, United States

## Abstract

Cardiovascular disease (CV) is a leading cause of death worldwide. Frailty is a growing geriatric syndrome. Limited research has explored the relationship between cardiovascular disease and frailty. This study assesses the association between a cardiovascular disease history and older adults’ frailty. We used data from the SABE (Salud, Bienestar y Envejecimiento) Colombia Study, a cross-sectional nationwide population-based survey (2015) including community-dwelling adults ≥60 years. A secondary dataset of 5,242 participant responses was analyzed. Frailty was assessed according to the Fried Frailty Phenotype (weakness, low speed, low physical activity, exhaustion, and weight loss) and categorized as frail (3 or more components) vs. not frail or pre-frail. We performed logistic regression analysis to assess the association between a history of cardiovascular disease (hypertension, stroke, heart disease) and frailty, adjusting for socio-demographics, other comorbidities, and functional status. The prevalence of cardiovascular disease was 59.4%, and frailty was 20.0%. Compared to those who do not have CV disease, those having 1 CV disease (OR=1.23, 95% CI 1.04-1.46) or ≥2 CV diseases (OR=1.55, 95% CI 1.24-1.94) were associated with higher odds for frailty. A higher number of CV diseases were independently associated with frailty among older Colombian adults. In future studies, preventing CV disease might help to decrease frailty in older adults.

